# Supplementary material for: Diversity and structure of prokaryotic communities within organic and conventional farming systems in central highlands of Kenya
Source: PLoS One. 2020 Aug 13;15(8):e0236574. doi: 10.1371/journal.pone.0236574 (PMC7425915; doi:10.1371/journal.pone.0236574)
Supplement: Attachment — Submitted filename: Response to reviewers.docx [file pone.0236574.s005.docx]

Prof. Gabriel Moreno-Hagelsieb,

Academic Editor
PLOS ONE

20^th^ May, 2020

Dear Gabriel Moreno-Hagelsieb,

**RE: REVIEWERS’ COMMENTS ON MANUSCRIPT PONE-D-19-32718 AND HOW THEY HAVE BEEN ADDRESSED**

I would like to appreciate this opportunity to resubmit a revised version of the manuscript entitled “Diversity and structure of prokaryotic communities within organic and conventional farming systems in central highlands of Kenya” to be considered for publication in PLOS ONE Journal as an original article. We appreciate the reviewers’ comments and have addressed the points raised during the review process fully to improve the manuscript for publication as follows;

**Academic Editor’s Comment 1**: If you would like to make changes to your financial disclosure, please include your updated statement in your cover letter.

**Response from Authors**: The authors would not like to make any changes to their financial disclosure.

**Academic Editor’s Comment 2**: To enhance the reproducibility of your results, we recommend that if applicable you deposit your laboratory protocols in [protocols.io](http://protocols.io/), where a protocol can be assigned its own identifier (DOI) such that it can be cited independently in the future. For instructions see:  <http://journals.plos.org/plosone/s/submission-guidelines#loc-laboratory-protocols>
**Response from Authors**: The study methodology used standard protocols as referenced within the manuscript. Therefore, the authors do not wish to deposit any protocol.

**Journal Requirements:**

When submitting your revision, we need you to address these additional requirements.

1. Please ensure that your manuscript meets PLOS ONE's style requirements, including those for file naming. The PLOS ONE style templates can be found at
<http://www.journals.plos.org/plosone/s/file?id=wjVg/PLOSOne_formatting_sample_main_body.pdf> and <http://www.journals.plos.org/plosone/s/file?id=ba62/PLOSOne_formatting_sample_title_authors_affiliations.pdf>

2. We note that Figure 1  in your submission contain [map/satellite] images which may be copyrighted. All PLOS content is published under the Creative Commons Attribution License (CC BY 4.0), which means that the manuscript, images, and Supporting Information files will be freely available online, and any third party is permitted to access, download, copy, distribute, and use these materials in any way, even commercially, with proper attribution. For these reasons, we cannot publish previously copyrighted maps or satellite images created using proprietary data, such as Google software (Google Maps, Street View, and Earth). For more information, see our copyright guidelines: <http://journals.plos.org/plosone/s/licenses-and-copyright>.

We require you to either (1) present written permission from the copyright holder to publish these figures specifically under the CC BY 4.0 license, or (2) remove the figures from your submission:
 a)    You may seek permission from the original copyright holder of Figure 1 to publish the content specifically under the CC BY 4.0 license.

We recommend that you contact the original copyright holder with the Content Permission Form (<http://journals.plos.org/plosone/s/file?id=7c09/content-permission-form.pdf>) and the following text:
“I request permission for the open-access journal PLOS ONE to publish XXX under the Creative Commons Attribution License (CCAL) CC BY 4.0 (<http://creativecommons.org/licenses/by/4.0/>). Please be aware that this license allows unrestricted use and distribution, even commercially, by third parties. Please reply and provide explicit written permission to publish XXX under a CC BY license and complete the attached form.”

Please upload the completed Content Permission Form or other proof of granted permissions as an "Other" file with your submission.

In the figure caption of the copyrighted figure, please include the following text: “Reprinted from [ref] under a CC BY license, with permission from [name of publisher], original copyright [original copyright year].”

b)    If you are unable to obtain permission from the original copyright holder to publish these figures under the CC BY 4.0 license or if the copyright holder’s requirements are incompatible with the CC BY 4.0 license, please either i) remove the figure or ii) supply a replacement figure that complies with the CC BY 4.0 license. Please check copyright information on all replacement figures and update the figure caption with source information. If applicable, please specify in the figure caption text when a figure is similar but not identical to the original image and is therefore for illustrative purposes only.

The following resources for replacing copyrighted map figures may be helpful: USGS National Map Viewer (public domain):  <http://viewer.nationalmap.gov/viewer/>
The Gateway to Astronaut Photography of Earth (public domain): <http://eol.jsc.nasa.gov/sseop/clickmap/>

Maps at the CIA (public domain): <https://www.cia.gov/library/publications/the-world-factbook/index.html> and <https://www.cia.gov/library/publications/cia-maps-publications/index.html>
NASA Earth Observatory (public domain): <http://earthobservatory.nasa.gov/>
Landsat: <http://landsat.visibleearth.nasa.gov/>
USGS EROS (Earth Resources Observatory and Science (EROS) Center) (public domain): [http://eros.usgs.gov/#](http://eros.usgs.gov/)
Natural Earth (public domain): <http://www.naturalearthdata.com/>

**Response from Authors:** Figure 1 has been removed from the manuscript on lines 84 – 87 since the sites are also indicated by the GPS coordinates.

**Reviewers' comments:** Reviewer's Responses to Questions
**Comments to the Author**
1. Is the manuscript technically sound, and do the data support the conclusions?

The manuscript must describe a technically sound piece of scientific research with data that supports the conclusions. Experiments must have been conducted rigorously, with appropriate controls, replication, and sample sizes. The conclusions must be drawn appropriately based on the data presented.

Reviewer #1: Partly

2. Has the statistical analysis been performed appropriately and rigorously?

Reviewer #1: Yes

3. Have the authors made all data underlying the findings in their manuscript fully available?

The [PLOS Data policy](http://www.plosone.org/static/policies.action#sharing) requires authors to make all data underlying the findings described in their manuscript fully available without restriction, with rare exception (please refer to the Data Availability Statement in the manuscript PDF file). The data should be provided as part of the manuscript or its supporting information, or deposited to a public repository. For example, in addition to summary statistics, the data points behind means, medians and variance measures should be available. If there are restrictions on publicly sharing data—e.g. participant privacy or use of data from a third party—those must be specified.

Reviewer #1: Yes

4. Is the manuscript presented in an intelligible fashion and written in Standard English?

PLOS ONE does not copyedit accepted manuscripts, so the language in submitted articles must be clear, correct, and unambiguous. Any typographical or grammatical errors should be corrected at revision, so please note any specific errors here.

Reviewer #1: No

**5. Review Comments to the Author**

Please use the space provided to explain your answers to the questions above. You may also include additional comments for the author, including concerns about dual publication, research ethics, or publication ethics. (Please upload your review as an attachment if it exceeds 20,000 characters)

**Reviewer #1: Review**

**Reviewer Comment 1**: The analysis of microbial diversity inhabiting the soil and in direct relation to the plants is an interesting approach to evaluate under two different farming systems. The authors explain their samples come from a long-term exposition under different fertilization practices, and at different nutrient input levels (low and high). However, different crop rotation is seen throughout the years, where the total amounts of nutrients used for each treatment also varies with time and season.
**Response from Authors**: OK

**Reviewer Comment 2**: Though the two different sites have similar geographical characteristics, such as altitude, temperature and cropping periods, soil type varies as well as the crops grown there.

**Response from Authors**: Crops grown in both sites are the same.

**Reviewer Comment 3**: Soil type and plant species are known as the most important factors in shaping bacterial communities. Thus, the structure of microbial community in each site is thought to be different even before the experiment begins. Most of the analysis and discussion is based in trying to find similarities or differences in the diversity of microbial communities between the two sites, but because of the reasons above, interesting comparisons would better come from Conventional versus Organic and the different nutrient levels, regardless the site. Thereby, differences in microbial diversity, would be meaningful as the discussion will be strictly focused in the functioning of each community structure and the effects of the farming system in the microbial diversity.

**Response from Authors**: In this study the comparison has been done at the following levels; (i) between farming systems (Organic versus Conventional) and the different nutrient levels, regardless of the site, (ii) at different input levels (high and low input levels) within and between sites. The sources of variation were System, Site and the interaction between System and Site. However, the diversity indicators within farming systems and sites showed no significant difference P>0.05. This is indicated within the manuscript in lines 223-224.It is important to note that high input farming systems were compared together while low input farming systems were compared together

**Reviewer Comment 4**: Also having an unfertilized plot would be a perfect control to compare both, physicochemical parameters and microbial diversity.

**Response from Authors**: The Long Term Farming System Experiment was designed as a system comparison trial within and between sites for a period of 20 years. There is no unfertilized plot since it was not considered as a farming system.

**Reviewer Comment 5**: If comparing organic versus conventional microbial diversity per site, a Venn diagram would be a good alternative, showing the numbers at any taxa level (maybe genus) would help to discuss shared and unique ones.

**Response from Authors**: Further analyses have been carried out at order taxa level. A Venn diagram for each data set in both sites has been added to the manuscript in Fig 3a, 3g, 4a and 4g. The shared and unique taxa at order level within each farming system have also been shown on the pie charts (Fig. 3b - f, 3h – l, 4b – f and 4h - k) in lines 198 – 221 within the manuscript.

**Reviewer Comment 6**: During the discussion, I can highlight a few more considerations/questions for this study:

High-throughput sequencing involved both 16S rDNA and 16S rRNA cDNA, but no more relevant differences in results or discussion is highlighted. What about the major differences of bacterial groups found in organic versus conventional farming? Were there some only found in one system but not in other worth to mention? Only organic microbial diversity is discussed.

**Response from Authors**: The results have been updated to show shared and unique prokaryotic taxa within 16S rDNA and 16S rRNA cDNA datasets in farming systems (lines 198 – 221). The discussion has also been enriched to cover both farming systems (line 292 – 323).

**Reviewer Comment 7**: Most of the discussion is focused on differences of number of OTUs, but not in relevant community structure for each type of agricultural management. Thus, differences in chemical properties are attributed to the inherent and particular soil composition.

**Response from Authors**: The community structure for each type of agricultural management system has been updated in the results section (lines 198 – 221). The discussion has also been enriched to cover the relevance of community structure in farming systems lines 285 – 319 and lines 351 – 359.

**Reviewer Comment 8**: The authors conclude that Conventional systems harbor a higher diversity than Organics in both sites. However, similar studies have found that agricultural practices such as fertilization and tillage reduce the diversity of soil organisms, and some found that microbial diversity increases in organic farms (Tsiafouli et al.2015; Zhang et al., 2019). Therefore, combining synthetic with natural products provoked the uncertainty in the results. I.e. Conv-High and Org-low showed similar richness and Shannon index values in Tika (16S rDNA), Conv-low and Org-High had the same diversity (6.09) as shown in the Shannon index (Table 3). Differences in unique OTUs differed in Chuka site, where Conventional levels had more than Organic; however, the diversity index was again very similar. An additional Anova test would help to clarify those differences among farming systems.

**Response from Authors**:

Combining synthetic inputs with natural products within conventional systems possibly aggravated the uncertainty in the results, i.e. Conv-High and Org-low showed similar richness and Shannon index values in Thika (16S rDNA), Conv-low and Org-High had the same diversity (6.09) as shown in the Shannon index (Table 3). Although there were OTU differences among farming systems as shown in Table 3, alpha diversity indices within farming systems and sites showed no significant difference (P>0.05) in Richness (S) and Shannon index (H’); (Lines 223-224). Similarly, there were no significant differences observed at Thika site (ANOSIM P<0.672 and 0.241 within 16S rDNA and 16S rRNA cDNA datasets, respectively); Lines 232-234. However, Analysis of Similarity pointed to highly significant differences between OTUs within high and low input farming systems (P<0.001) at Chuka site Lines 230-232.

**Reviewer Comment 9**: Finding the unique OTUs could be key step in the analysis. The authors explained after filtering and trimming the sequences, they were clustered at 97%. This conventional step could have better results if eliminating exactly same sequences (dereplication) and counting all the remaining ones as true OTUs, to avoid underestimate some species.

**Response from Authors**: The FASTQ sequences were demultiplexed and quality checked using a pipeline that denoises sequences, removes chimeras, picks representative sequences, calculates denoising statistics and creates an OTU table. During this step, sequences which were <200 base pairs after phred20- base quality trimming, sequences with ambiguous base calls, and those with homopolymer runs exceeding 6bp were removed. At this step, any exact same sequences (dereplication) were removed. The denoising statistics indicated satisfactory level of sequence quality and there was adequate representation of all high-quality sequences within the samples.

**Reviewer Comment 10**:

Finally, the conclusion (line 327) is that conventional farming support (include more) diverse prokaryotic communities compared to organic farming systems. It is important to discuss if having more or less diversity would be more or less favourable in terms of farming sustainability since very recently studies support that having a more even community play an important role in soil ecosystem health, compared to richness.

**Response from Authors**: The conclusion has been enriched appropriately in lines 361-376.

**Academic Editor’s Comment 2**: While revising your submission, please upload your figure files to the Preflight Analysis and Conversion Engine (PACE) digital diagnostic tool, <https://pacev2.apexcovantage.com/>. PACE helps ensure that figures meet PLOS requirements. To use PACE, you must first register as a user. Registration is free. Then, login and navigate to the UPLOAD tab, where you will find detailed instructions on how to use the tool. If you encounter any issues or have any questions when using PACE, please email us at [figures@plos.org](mailto:figures@plos.org). Please note that Supporting Information files do not need this step.

**Response from Authors**: All figures have been uploaded to the Preflight Analysis and Conversion Engine (PACE) digital diagnostic tool and the corrected figures have been filed for use in this manuscript. However, there were no PACE adjustments on pie diagrams uploaded on PACE.

We believe to have adequately responded on all the comments raised regarding this manuscript in readiness for its publication to your journal.

Thank you for your consideration.

Yours Sincerely

Dr. Romano Mwirichia,

Department of Biological Sciences, University of Embu,

P.O Box 6 Embu.
