# Supplementary material for: Diversity and structure of prokaryotic communities within organic and conventional farming systems in central highlands of Kenya
Source: PLoS One. 2020 Aug 13;15(8):e0236574. doi: 10.1371/journal.pone.0236574 (PMC7425915; doi:10.1371/journal.pone.0236574)
Supplement: Attachment — Submitted filename: Response to Reviewers.doc [file pone.0236574.s006.doc]

Prof. Gabriel Moreno-Hagelsieb,

Academic Editor
PLOS ONE

24^th^ June, 2020

Dear Gabriel Moreno-Hagelsieb,

**RE: REVIEWERS’ COMMENTS ON MANUSCRIPT PONE-D-19-32718 AND HOW THEY HAVE BEEN ADDRESSED**

I would like to appreciate this opportunity to resubmit a revised version of the manuscript entitled “Diversity and structure of prokaryotic communities within organic and conventional farming systems in central highlands of Kenya” to be considered for publication in PLOS ONE Journal as an original article. We appreciate the reviewers’ comments and have addressed the points raised during the review process fully to improve the manuscript for publication as follows;

**Reviewer #1:**

**Comments to the Author**

**Reviewer Comment 1**: In your discussion you clearly express that conventional farming systems supported significantly higher species richness in both sites, which in fact was not supported by any of the index, meaning no significant difference in Richness and Shannon index. The only difference was seen between OTUs within high and low input farming systems at Chuka.

**Response from Authors**: The sentence has been rephrased to reflect the results (Lines 304-309).

**Reviewer Comment 2**: Information provided for Thika taxonomical orders differ between the results. In Fig 3, 213 taxa (Order) are supposed to be shown, but the Venn diagram contains 229.

**Response from Authors**: Thika taxonomical orders are 229 as shown in the Venn diagram. This correction has been effected in line 179. We apologize for the typing error.

**Reviewer Comment 3**: Furthermore, only 62 orders are shown in S3 Table, which corresponds to the list provided for this site. On the other hand, there is an accurate correspondence of these information for Chuka site.

**Response from Authors**: During data analysis, all the samples that scored a minimum relative abundance of 5 % and above in at least one (1) sample per farming system were selected for diagram presentation. All samples scoring below 5 % across all farming systems were reported in the narrative in lines 170 - 184. Therefore, in supplementary table S3, sixty two (62) orders had scored at least 5% in one sample within the farming system.

**Reviewer Comment 4**: What is the order of pie charts? It is not mention in the figure legend.

**Response from Authors**: The pie charts were made at order level. This has been added to the figure legend.

**Reviewer Comment 5**: Please review: Line 57: hence,

**Response from Authors**: A comma has been added after the word “hence” in Line 57.

**Reviewer Comment 6**: Please review: Line 312: to16S

**Response from Authors**: A space has been added to separate “to16S” in Line 315.

**Reviewer Comment 7**: Please review: Line 294: the idea is not clear, maybe substitute effect for affect.

**Response from Authors**: The sentence has been rephrased to make it clear.

**Reviewer Comment 8.** Have the authors made all data underlying the findings in their manuscript fully available? The PLOS Data policy requires authors to make all data underlying the findings described in their manuscript fully available without restriction, with rare exception (please refer to the Data Availability Statement in the manuscript PDF file). The data should be provided as part of the manuscript or its supporting information, or deposited to a public repository. For example, in addition to summary statistics, the data points behind means, medians and variance measures should be available. If there are restrictions on publicly sharing data—e.g. participant privacy or use of data from a third party—those must be specified.

**Response from Authors**: The sequences were submitted to NCBI Sequence Read Archive. The SRA accession numbers are as indicated in lines 146-150. Statistical data analysis summaries for soil physicochemical characteristics were as shown in Table 4. Analysis of variance (ANOVA) was performed at P < 0.05, 0.01 and 0.001 using a linear mixed-effect model with farming system and site as fixed effects, while replication was used as random effect. Least mean squares were computed and means separated using Tukey’s ad hoc method. Letters *a-d* were used to designate significant differences between means at P ≤ 0.05. The authors have availed the soil physicochemical raw data as an attachment for purposes of information in order to show the values behind means variance measures.

We believe to have adequately responded on all the comments raised regarding this manuscript in readiness for its publication in your esteemed journal.

Thank you for your consideration.

Yours Sincerely

Dr. Romano Mwirichia,

Department of Biological Sciences, University of Embu,

P.O Box 6 Embu.
